# Bilateral Phacoemulsification in an African Elephant (*Loxodonta africana*)

**DOI:** 10.1155/2019/2506263

**Published:** 2019-07-10

**Authors:** Anthony J. Cerreta, Richard J. McMullen Jr, Heather E. Scott, Jennifer D. Ringenberg, Julie E. Hempstead, Ryan S. DeVoe, Michael R. Loomis, Larry J. Minter

**Affiliations:** ^1^Department of Clinical Sciences, North Carolina State University, College of Veterinary Medicine, 1060 William Moore Drive, Raleigh, NC 27607, USA; ^2^Auburn University College of Veterinary Medicine, JT Vaughan Large Animal Teaching Hospital, 1500 Wire Road, Auburn, AL 36849, USA; ^3^Hanes Veterinary Medical Center, North Carolina Zoo, 4401 Zoo Parkway, Asheboro, NC 27205, USA; ^4^Blue Pearl Specialty and Emergency Pet Hospital, 3000 Busch Lake Blvd., Tampa, FL 33614, USA; ^5^Gulf Coast Veterinary Specialists, 8042 Katy Freeway, Houston, TX 77024, USA; ^6^Department of Animal Health, Disney's Animal Kingdom, 2901 Osceola Parkway, Lake Buena Vista, FL 32830, USA

## Abstract

A 37-year-old bull African elephant (*Loxodonta africana*) at the North Carolina Zoo (NCZ) was diagnosed with bilateral cataracts leading to behavioral changes and significant weight loss secondary to functional blindness. On initial examination, a weight loss of 234 kg, a mature cataract in the right eye, and a focal cataract in the left eye were diagnosed. Ultrasound and electroretinography (ERG) indicated normal retinal attachment and both eyes were viable candidates for surgery. After careful planning and behavioral training, the left cataract was surgically removed via phacoemulsification and irrigation/aspiration. The right eye subsequently developed a ventral lens subluxation, and phacoemulsification and irrigation/aspiration were performed six months after the first procedure. Four years after surgery, menace response, palpebral reflex, dazzle reflex, and pupillary light reflexes were present in both eyes. Body weight was 5,515 kg, 88kg more than at the time of the second surgery. This is the first published report of an African bull elephant undergoing bilateral cataract removal using phacoemulsification and irrigation/aspiration. The lack of significant postoperative inflammation and uneventful recovery of the elephant suggests that this surgical procedure along with proper preoperative planning and postoperative medical management can be a safe and effective treatment option for elephants with cataracts.

## 1. Introduction

Lenticular abnormalities such as cataracts and lens luxation have been described in both Asian and African elephants [1, 2]. However, the extent of ocular disease in these species is equivocal due to limited data from ocular examination and treatment [3]. It has been proposed that excess infrared and visible radiation due to limited shade and reflection of heat from the ground are responsible for lesions of the lens and retina [4]. These risk factors place both wild elephants and those in human care at an increased chance for ocular damage. In field studies of Asian and African elephants, large proportions of the populations had ocular lesions, including cataracts [1, 4–6]. Surgical intervention is not considered necessary until cataract maturation affects sight and quality of life [7, 8]. This is especially relevant in zoological species where the risks of anesthesia and the ability to administer pre- and postoperative treatments must be considered when deciding whether to pursue surgical correction of cataracts.

Several methods used to surgically address cataracts in humans, wildlife, and domestic animals have been reported [9–13]. Phacoemulsification is the preferred method for cataract removal in domestic species, even in animals with large eyes, including the horse, because it allows for a smaller corneal incision and thus a faster return to activity [14, 15]. Phacoemulsification requires that a small incision be made in the cornea for insertion of a handpiece to ultrasonically fragment the lens, which is then aspirated for removal [8]. Furthermore, phacoemulsification can be used for all stages of cataract maturity and has a favorable prognosis for restoring vision [16]. Cataract removal via phacoemulsification has been anecdotally reported in one Asian elephant, although details of the procedure were not provided [3].

## 2. Case Presentation

### 2.1. Initial Ophthalmic Examination

Keepers at the North Carolina Zoo (NCZ) reported that a 37-year-old, bull African elephant (*Loxodonta africana*) appeared to have cloudiness in both eyes in March 2010 with a concomitant weight loss of 188 kg. Complete ophthalmic examination was performed which indicated that direct and consensual pupillary light reflexes (PLR), palpebral reflexes, and dazzle reflexes were present and there was no fluorescein stain uptake in either eye. Slit lamp biomicroscopy of the anterior chamber (AC) was within normal limits, with the exception of a focal cataract in the right eye and a newly forming incipient cataract in the left eye. Indirect ophthalmoscopy revealed no abnormalities. 0.03% flurbiprofen sodium ophthalmic solution (Bausch and Lomb, Bridgewater, NJ, 08807) was prescribed topically twice daily indefinitely to decrease the inflammation associated with cataract formation.

**Figure 1:**
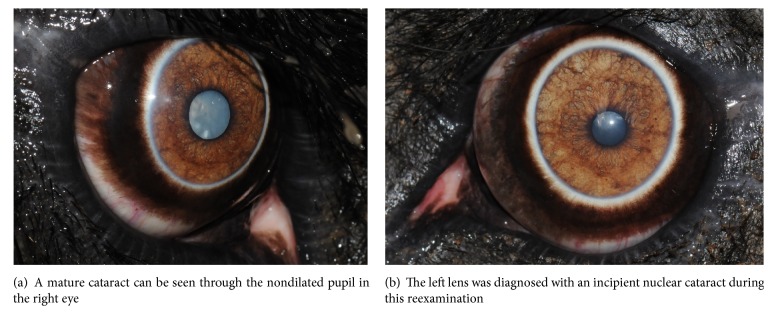
Ophthalmic reexamination six months after the initial examination in 2010.

A complete ophthalmic examination was repeated six months later. The size of the cataracts had increased in both eyes and the elephant had an additional weight loss of 89 kg. A mature cataract was found in the right eye (Figure 1(a)) and a nuclear incipient cataract was found in the left eye (Figure 1(b)). Intraocular pressures (IOP) measured via applanation tonometry (Tono-Pen Vet™, Dan Scott and Associates, Inc., Westerville, OH) were 18 mmHg in the right eye and 21 mmHg in the left eye which are considered to be within normal limits [17, 18]. Based upon examination, it was suspected that there was only limited vision in the right eye and there were only light and shadows visible in the left eye. The elephant began showing signs of significant blindness such as unsure footing, knocking his tusks into doorways, and utilizing his trunk to navigate. Approximately one year after initial diagnosis, he was no longer able to independently traverse the runway from the holding barn to the habitat. In addition to the ophthalmic abnormalities, an additional weight loss of 269 kg was noted. Outdoor paddocks and indoor stalls were available to the elephant during the day, but the majority of the time he was observed leaning against supports or swaying in place. A total weight loss of 546 kg, the bull's loss of vision, and development of subsequent stereotypical behavior prompted discussion about his quality of life and the feasibility of performing surgery to remove his cataracts.

### 2.2. Ancillary Diagnostic Tests

**Figure 2:**
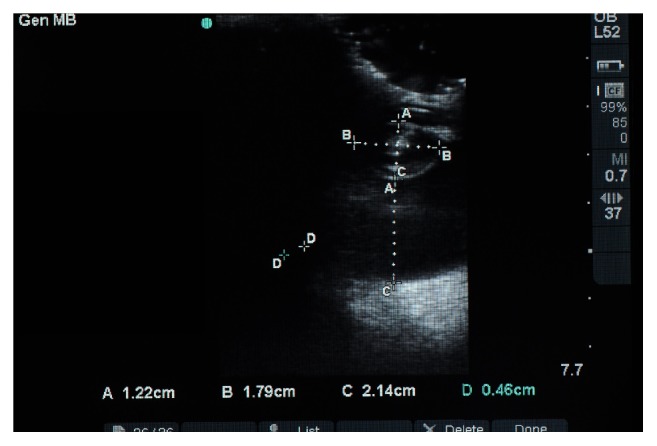
B-mode ocular ultrasonography of the elephant's right eye revealing a hyperechoic nuclear lens. Despite the clinical diagnosis of a mature cataract, the lens cortex appears to be less significantly affected in the ultrasound image. The crystalline lens thickness (A: 12.2 mm), crystalline lens diameter (B: 17.9 mm), and the vitreal chamber depth (C: 21.4 mm) are visible in this image, as well. Note that D is not a valid measurement and that the caliper measurements were inadvertently saved.

Ocular ultrasonography and electroretinography were performed to determine whether the elephant was a candidate for phacoemulsification and intraocular lens (IOL) implantation. Transcorneal B-scan ultrasonography revealed hyperechoic lens in both eyes, consistent with cataractous changes (Figure 2). Preoperative anterior chamber depth (ACD) measured 5.0 mm, axial globe length measured 38.6 mm, and crystalline lens thickness measured 12.2 mm. Electroretinography (ERG), performed in the darkened elephant holding area, confirmed that the retina of the right eye (OD) was functional; however, due to waning cooperation by the patient, ERG was unable to be performed in the left eye (OS). Both the dazzle reflex and PLRs were positive in both eyes (OU). The results of a complete blood count and serum biochemistry panel were within normal limits for an adult elephant [19]. Based on these preoperative findings, the elephant was considered a good candidate for surgery.

### 2.3. Treatment and Surgical Management

Unilateral phacoemulsification was chosen to remove the mature cataract in the right eye. Preoperative treatment with topical ophthalmic medications was implemented 24 hours prior to surgery. Medications were applied with a 1.0 ml syringe with the needless hub at a volume of 0.1 ml. These included 1.0% prednisolone acetate ophthalmic suspension (Omnipred, Alcon Laboratories, Inc., Fort Worth, Texas 76134) (q 30 minutes) to prevent endophthalmitis and reduce intraocular inflammation, 0.5% moxifloxacin hydrochloride ophthalmic solution (Vigamox, Alcon Laboratories, Inc., Fort Worth, Texas 76134) (q 30 minutes) to prevent endophthalmitis, and 1.0% nepafenac ophthalmic suspension (Nevanac, Alcon Laboratories, Inc., Fort Worth, Texas 76134) (q 12 hours) to control intraocular inflammation prior to surgery.

**Figure 3:**
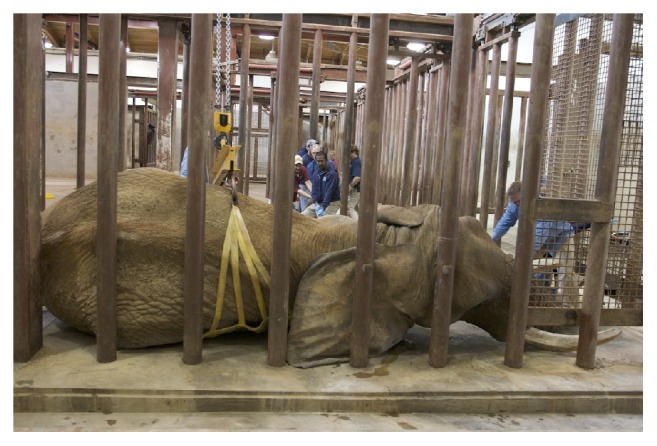
The original plan was to perform surgery on the right eye, if possible, but the elephant went into right lateral recumbency and could not be maneuvered into left lateral recumbency. As a result, surgery was performed on his left eye.

**Figure 4:**
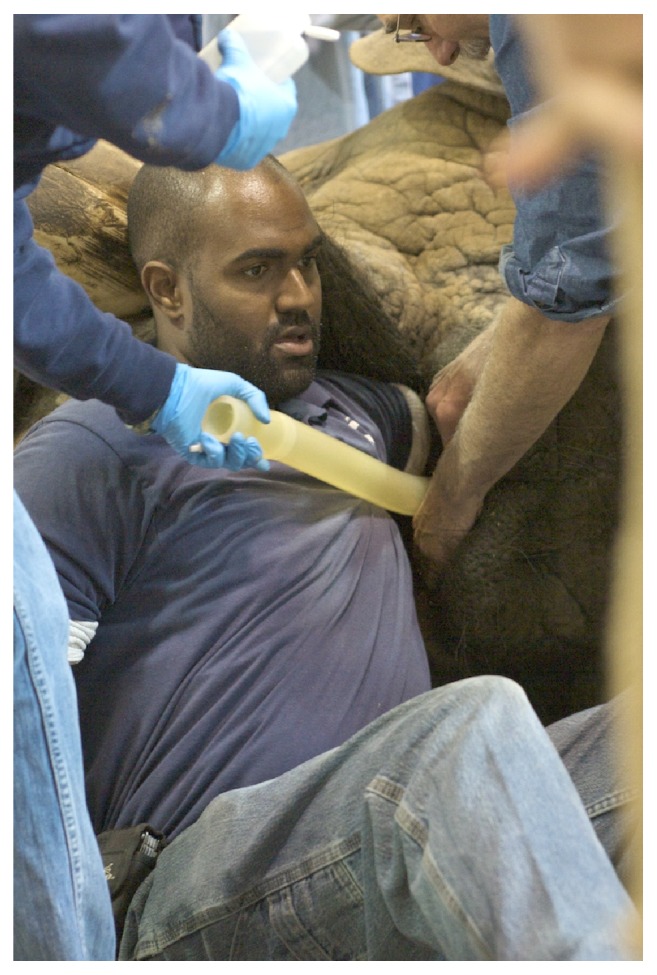
A size 30 endotracheal tube is being placed, under manual control, along the left arm of this veterinarian.

**Figure 5:**
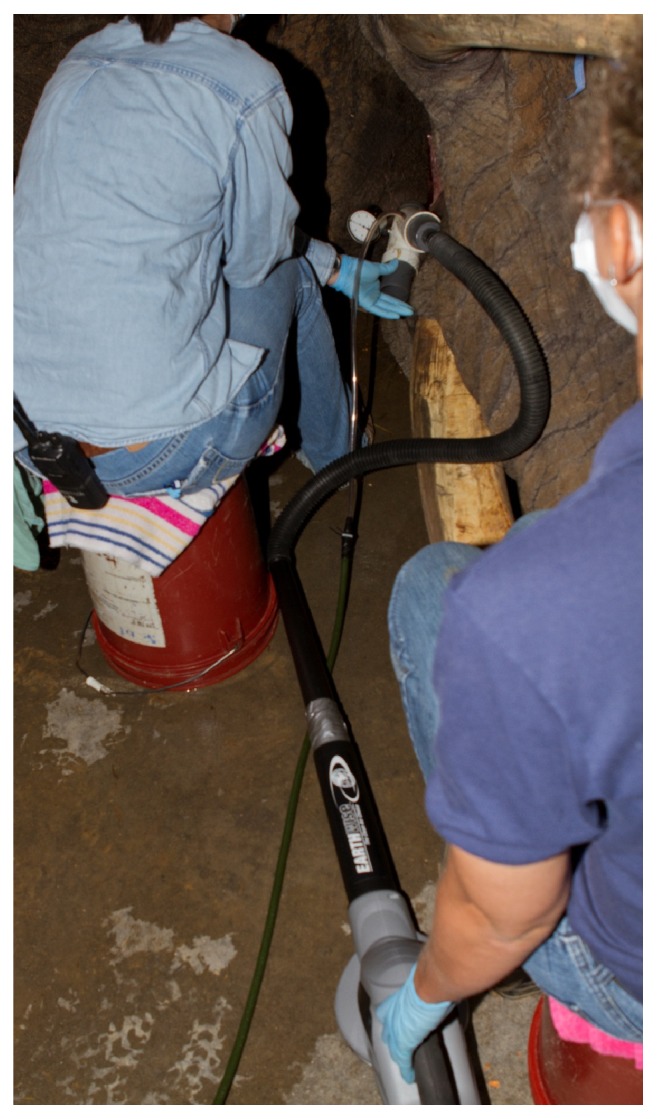
A modified Y-piece apparatus and electric leaf blower were attached to the endotracheal tube to provide intermittent positive pressure ventilation (IPPV) and supplemental oxygen to the elephant bull during the duration of the anesthesia.

The elephant was induced with etorphine (1.5 *μ*g/kg, IV) and became semisternally recumbent within 2 minutes. The elephant could not be manually manipulated into left lateral recumbency. However, right lateral recumbency was achieved (Figure 3) and the decision to continue with phacoemulsification of the left eye, rather than the right eye, was made. Six mg of etorphine was added to a 250 ml bag of 0.9% sodium chloride and a constant rate infusion was instituted at 3 ml/hour for the first 30 minutes of the procedure, increased to 6 ml/hour for one hour, and then reduced to 4 ml/hour for the remainder of the procedure. A size 30 endotracheal tube (ET) was used for intubation (Figure 4). Intermittent positive pressure ventilation (IPPV) and supplemental oxygen were provided with a modified Y-piece apparatus and electric leaf blower (Figure 5) [20]. This apparatus was capable of maintaining blood oxygen saturation (SPO_2_) levels of 93-99% throughout the procedure.

**Figure 6:**
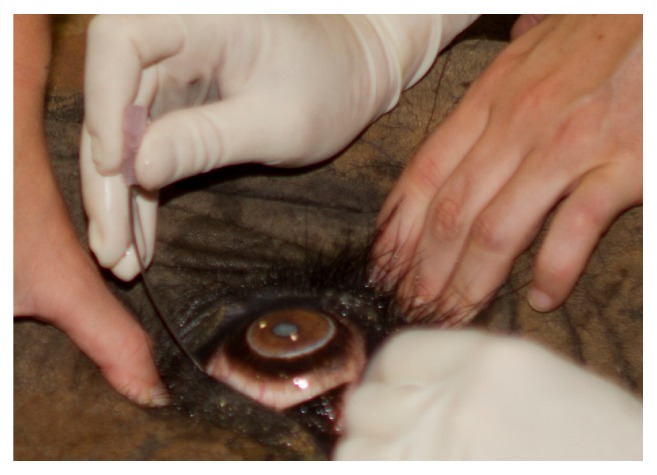
A modified retrobulbar block was performed using a slightly bent, 19-gauge, 2.5-inch needle to deposit approximately 25 ml of 2% mepivacaine (Pharmacia & Upjohn Company, Pfizer Inc. NY, NY, USA) through the ventral and dorsal bulbar conjunctiva behind the globe.

**Figure 7:**
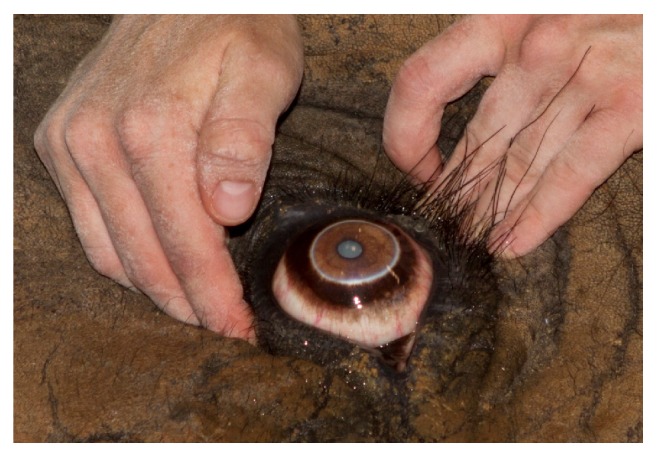
Manual digital pressure was applied simultaneously to the upper and lower eyelids to displace the globe anteriorly, thereby providing better access to the sites of injection for the modified retrobulbar block described and shown in Figure 6.

A modified retrobulbar block was performed following aseptic preparation of the periocular region and bulbar conjunctiva. Using a 19-gauge, 2.5-inch spinal needle with a slight bend, approximately 25 ml of 2% mepivacaine (Pharmacia & Upjohn Company, Pfizer Inc. NY, NY, USA) was deposited behind the globe from both dorsal and temporal conjunctival access points (Figure 6) [21]. Access to these regions was facilitated by slight manual depression of the upper and lower eyelids (Figure 7).

**Figure 8:**
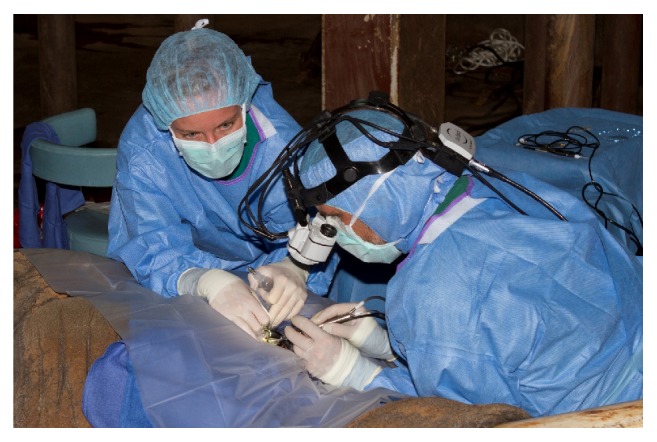
Due to the unconventional surgical location and positioning of the elephant, a head-mounted microscope (Varioscope M5®, Acrivet, Veterinary Division of S&V Technologies AG, Hennigsdorf, Germany) was utilized to facilitate visualization for the procedures.

**Figure 9:**
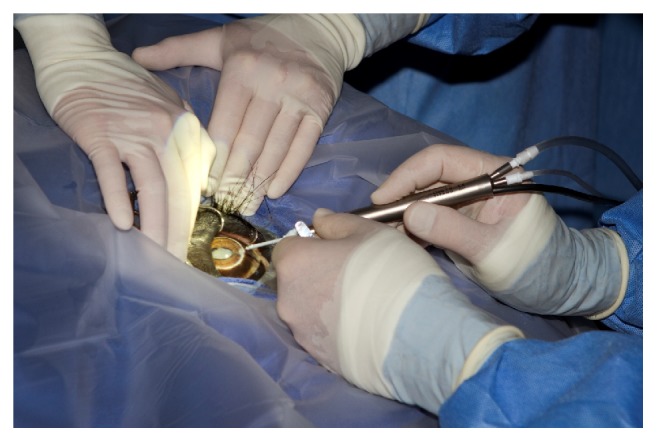
Phacoemulsification was performed using a 45-degree, 4 cm long handpiece designed specifically for horses (Acrivet, Veterinary Division of S&V Technologies AG, Hennigsdorf, Germany).

Due to the unconventional head position and limited access to the globe, a head-mounted microscope (Varioscope M5®, Acrivet, Veterinary Division of S&V Technologies AG, Hennigsdorf, Germany) was utilized (Figure 8). A trilaminar, clear corneal incision (CCI) was created [22–24]. Briefly, a 4 mm groove incision was created approximately 1 mm from the limbus at the 7 o'clock position using a 2.8 mm sapphire crescent blade (Acrivet, Veterinary Division of S&V Technologies AG, Hennigsdorf, Germany) and was followed by a 2 mm tunnel incision extending axially from a depth of approximately 50 percent of the initial groove incision. Finally, using a 2.8 mm sapphire slit/phaco blade (Acrivet, Veterinary Division of S&V Technologies AG, Hennigsdorf, Germany) entering through the tunnel incision, the deep corneal layers were penetrated, providing access to the anterior chamber through a hinged, self-sealing incision. 0.2-0.3 mL epinephrine hydrochloride was infused into the AC to facilitate pupil dilation and to control intraocular hemorrhage. Viscoelastic material was then used to fill the AC (Acrivet Biovisc 1.2% hyaluronic acid, Acrivet, Hennigsdorf, Germany). High frequency diathermy was used to create an anterior capsulotomy of approximately 11 mm in diameter [25]. A second stab incision was created at the 10 o'clock position using a 21-gauge needle to facilitate access of a second instrument. Phacoemulsification with a 45-degree, 4 cm equine-specific system was utilized to remove the cataract (Figure 9) [24, 26, 27]. A nucleus rotator with y-shaped tip (Acrivet, Veterinary Division of S&V Technologies AG, Hennigsdorf, Germany) was introduced, as necessary, to facilitate lens removal. Significant lens instability and an equatorial lens capsule rupture, which became evident during the initial stages of phacoemulsification, precluded implantation of an intraocular lens (IOL). The entire lens capsule was removed by use of a 17 mm, curved, capsulorhexis gripper (Acrivet, Veterinary Division of S&V Technologies AG, Hennigsdorf, Germany) with no resistance. The AC was reinflated with viscoelastic material, and the corneal incision was closed by use of 8-0 polyglactin (Polyglactin 910, Ethicon Inc., Somerville, NJ) in a reverse sawtooth suture pattern.

**Figure 10:**
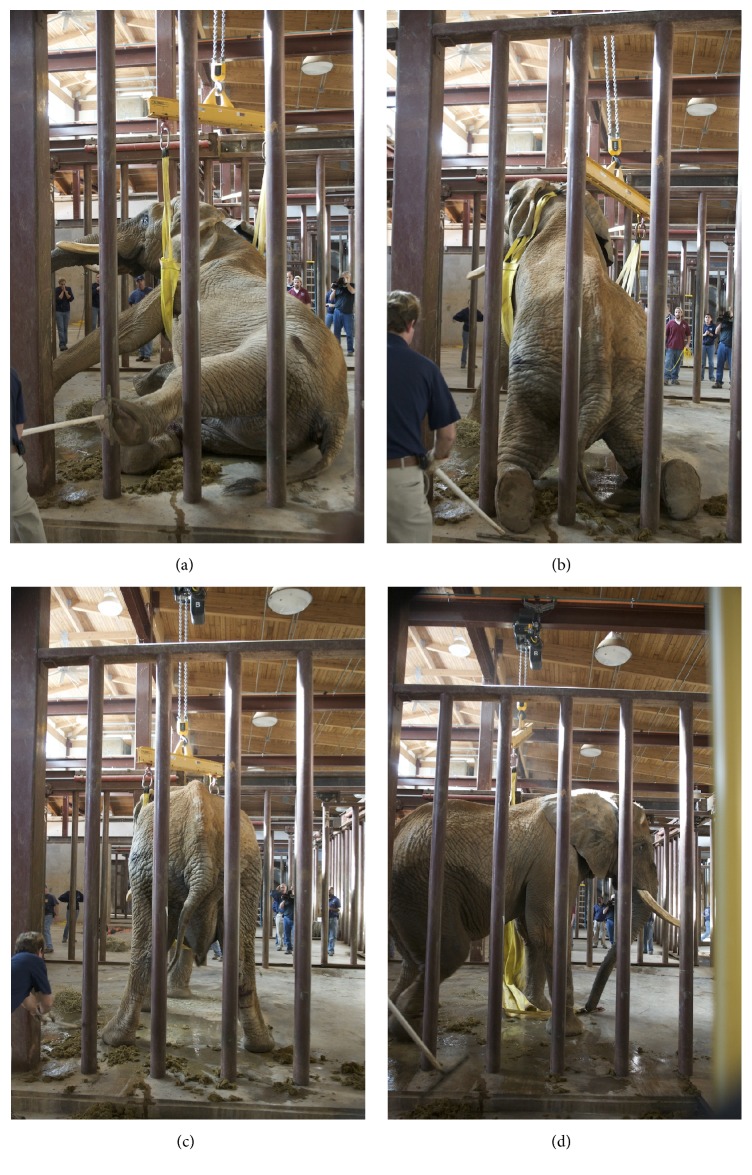
A series of images illustrating the use of an overhead hoist attached to a sling that was placed under his thorax prior to induction (see Figure 3).

The elephant was given naltrexone (500 mg IM, 500 mg IV) to reverse the effects of the etorphine and extubated. The animal became aroused and began moving his head within two minutes of receiving the injection. He was moderately disoriented and unable to rise on his own, requiring assistance with an overhead hoist (Figures 10(a)–10(d)). Perioperatively, the elephant received 13.5 L of lactated ringers solution IV and flunixin meglumine (500 mg IV, 500 mg subcutaneously). The remainder of recovery was uneventful and the elephant was confined to a stall for one month. Postoperative topical medical therapy of the left eye included prednisolone acetate (q 8 hours for 14 days), nepafenac ophthalmic suspension (q 12 hours for 14 days), and moxifloxacin hydrochloride ophthalmic solution (q 6 hours).

The elephant improved in attitude and activity level immediately following surgery and was able to navigate the holding area without issue, despite his being more than 9.00 diopters (D) hyperopic due to his being left aphakic postoperatively. All ophthalmic treatments were continued, as prescribed, for a total of 30 days. Thereafter, the medication frequencies were serially decreased at two-week intervals until they were discontinued 16 weeks after surgery. Due to the bull's continued increase in appetite and improved ability to navigate the area in the barn, he was returned to the habitat approximately one month postoperatively. Three months after the initial surgery in the left eye, a full ophthalmic exam was performed. The left eye appeared to be stable and healing well after recovery. Ocular ultrasound of the right eye showed that the cataract had progressed and a ventral lens subluxation had developed. Topical therapy to constrict the pupil and prevent increased intraocular pressure of the right eye included latanoprost 0.01% ophthalmic solution (Bausch and Lomb, Bridgewater, NJ, 08807) (q 6 hours for 30 days) and pilocarpine hydrochloride 2% solution (Akorn Animal Health, Lake Forest, IL 60045) subconjunctivally (q 6 hours until time of surgery).

Preoperative topical medications were started three days prior to the planned phacoemulsification of the right eye. These included 1.0% prednisone acetate ophthalmic solution (q 12 hours) to prevent endophthalmitis and reduce intraocular inflammation, 0.5% moxifloxacin hydrochloride ophthalmic solution (q 8 hours) to prevent endophthalmitis, and 1.0% nepafenac ophthalmic suspension (q 12 hours) to control intraocular inflammation prior to surgery.

**Figure 11:**
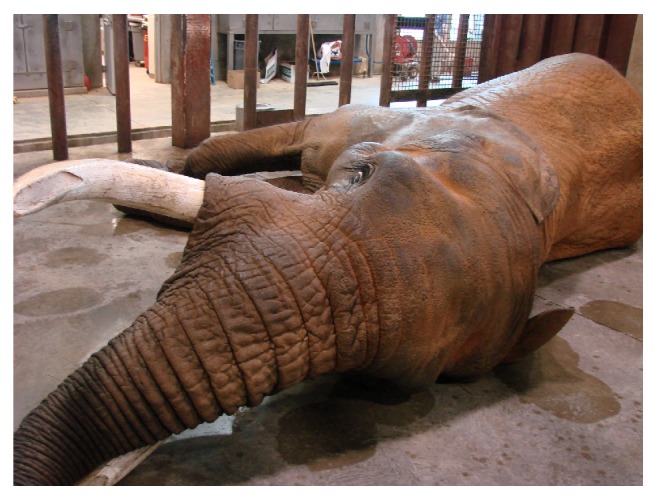
The elephant bull was photographed during a training session where he was practicing to lie down in left lateral recumbency on command, with the hope that he would end up in the appropriate position to perform phacoemulsification of his right eye.

To prevent the elephant from going down into right lateral recumbency, it was trained to stretch out in left lateral recumbency (Figure 11). Ten mg thiafentanil was administered via an 18-gauge butterfly catheter through an auricular vein in the right ear. The elephant stood up but fell back into left lateral recumbency within one minute of induction. The elephant was intubated with a 30 mm endotracheal tube and breathing was controlled via the same leaf blower apparatus described previously (Figures 4 and 5). A constant rate infusion of thiafentanil (mixed to a concentration of 0.1 mg/ml in 0.9% NaCl) was administered at a rate of 100 ml/hour IV. The phacoemulsification of the right eye and recovery from anesthesia proceeded in the same manner as described previously and proceeded without complication.

### 2.4. Outcome and Follow-Up

The elephant was examined two weeks postoperatively and a complete ophthalmic examination was performed. Menace response, palpebral reflex, dazzle reflex, and pupillary light reflexes were present in both eyes. IOP in both eyes were within normal limits and the incision sites appeared to be healing well. The elephant was cleared to be let out into his habitat but was started on atropine ophthalmic solution (Akorn Animal Health, Lake Forest, IL 60045) 1 drop q 12 hours for 3 days.

**Figure 12:**
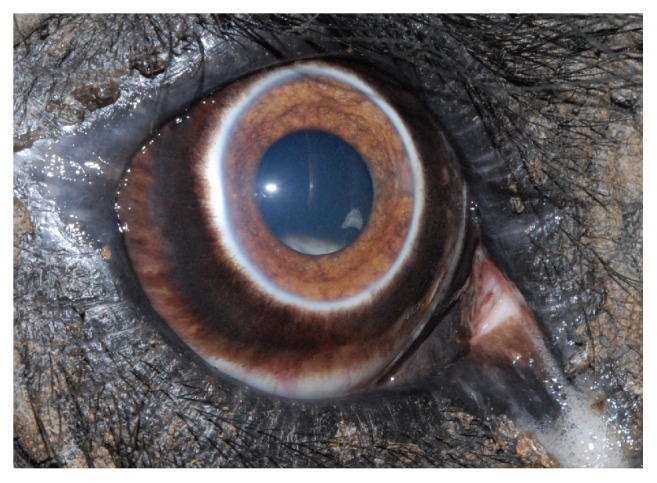
A small piece of what appears to be lens nuclear material can be visualized within the ventromedial pupil at approximately the 4 o'clock position.

**Figure 13:**
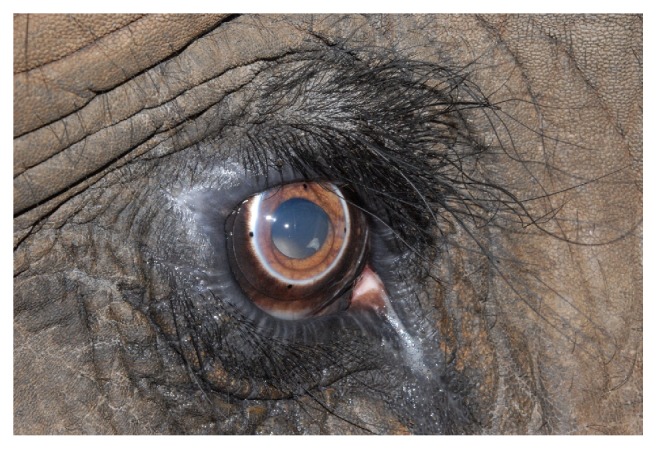
A corrective contact lens (+ 9.00 diopters) has been placed in the elephant's right eye in an attempt to correct the marked hyperopia due to his being left aphakic (Acrivet, Veterinary Division of S&V Technologies AG, Hennigsdorf, Germany). Note the smaller circular area within the center of the lens (this is most readily visible along the ventromedial pupil extending from approximately 2:30 to 9:00 o'clock) that represents the corrective portion of the lens. The four black dots at the 12, 3, 6, and 9 o'clock edges of the corrective contact lens are to make it easier to visualize during subsequent examinations.

Complete ophthalmic examinations were repeated at 3 weeks, 8 weeks, 12 weeks, 6 months, and 12 months. At the 8-week recheck, a small lens remnant was discovered in the vitreous in the right eye that could only be visualized when the pupil was fully dilated (Figure 12). As there were no signs of inflammation associated with the lens remnant, no action was taken at this time. Six months postoperatively, a corrective contact lens (Acrivet, Veterinary Division of S&V Technologies AG, Hennigsdorf, Germany) was placed in the right eye (Figure 13). Due to waning cooperation of the elephant, a corrective lens was not placed in the left eye. Twenty four hours later it was discovered that the corrective lens was no longer in place and was not replaced. The NCZ keepers reported that the elephant was behaving normally in his habitat and noted a marked improvement in his vision and body condition score, with a weight gain of 107 kg. Mild corneal edema in the right eye was discovered at the 12-month recheck and the left eye showed no active problems. Both aphakic globes were significantly hyperopic following surgery. Despite this, the elephant bull appeared to navigate his surroundings and was reintegrated into the herd. Four years postoperatively, his keepers noted only mild visual impairment and a normal body condition score for a bull elephant of his age. His weight was increasing at 5,515 kg, 88 kg more than at the time of the second surgery, and closer to his historical weight of 6,095 kg. He no longer displayed any evidence of stereotypical behavior and had successfully been integrated back into the herd, thus appearing to have a vastly improved quality of life following surgery.

## 3. Discussion

Cataracts are a common finding in many domestic species and are occasionally seen in African elephants (*Loxodonta africana*) [4, 9]. Cataracts form when there is a disruption to the normal anatomy of the lens and are often caused by trauma, disease, or age-related changes [28]. Cataract development in this elephant was suspected to be most likely due to age, with subsequent progression being relatively fast following diagnosis. There was no inflammation observed at the time of diagnosis or during the months (OS) or years (OD) prior to surgery. As a result, it was suspected that postoperative inflammation would be minimal following surgical removal of the lens(es). Preoperative functional retinal evaluation via ERG and ocular biometry via ultrasound were performed to determine if and which power intraocular lens implant could be used following removal of the cataractous lens via phacoemulsification and irrigation/aspiration. However, the significant lens instability (first noted following the initial stages of surgery, i.e., during creation of the anterior capsulorhexis) prevented implantation of an intraocular lens (IOL) implant.

Previously published methods for cataract removal in domestic and nondomestic species include lensectomy and phacofragmentation. A lensectomy, where the entire lens is removed, often requires multiple or larger incisions, which increases the potential damage to the anterior and posterior capsules and therefore exacerbates postsurgical complications in healing and recuperation [29]. Phacoemulsification employs ultrasound waves to break down, or emulsify, the lens with a high frequency ultrasound probe and then aspiration of the cortex and nucleus through a single needle [30]. Small‐incision phacoemulsification with aspiration utilizing instrumentation specifically modified for the equine eye is the current standard of care for cataract surgery in the horse, the species which this surgery was modeled after [16, 31–36]. Phacoemulsification and aspiration are most commonly performed through a single incision, utilizing a single needle to infuse irrigating solution, aspirate, and deliver ultrasonic phacoemulsification [15].

Utilizing a trilaminar (three‐step) corneal incision, whereby a proximal hinge is created at the base of the incision, helps to prevent the incision from leaking. This technique is advantageous in the horse, since it prevents the iris/corpora nigra from migrating to the corneal incision during phacoemulsification of the lens and aspiration of the cortex [36]. The trilaminar corneal incision also enables the anterior chamber to remain formed throughout the surgical procedure, thus reducing the amount of viscoelastic utilized throughout the surgery. This procedure also requires a small surgical incision, which minimizes the risk of intraoperative iris prolapse and results in fewer postoperative complications and quicker recovery [30, 36].

Following bilateral phacoemulsification and irrigation/aspiration, the elephant bull was able to navigate his surroundings, forage normally, and be reintegrated into the herd, thereby improving his quality of life. Four years postoperatively, the elephant had mild visual impairment, but a normal attitude and mentation for an adult bull elephant. He weighed 5,515 kg, 88kg more than at the time of the second surgery, and attained a normal body condition score for a bull elephant of his age. The rapid postoperative recovery of the elephant following phacoemulsification and his quick acclimation to navigating and foraging after recovery indicated that this surgical procedure, with proper planning and execution, can be a safe and effective treatment for cataracts in adult elephants. Regular ophthalmic examination in elephants should be included in their annual health check program. Early detection and treatment of any ocular abnormality may avoid the development of subsequent irreversible ocular pathology.
